# Identification of causative agents of infective endocarditis by metagenomic next-generation sequencing of resected valves

**DOI:** 10.3389/fcimb.2025.1532257

**Published:** 2025-03-13

**Authors:** Vladimir Lazarevic, Nadia Gaïa, Truong-Thanh Pham, Mikaël de Lorenzi-Tognon, Myriam Girard, Florian Mauffrey, Yannick Charretier, Gesuele Renzi, Christoph Huber, Jacques Schrenzel

**Affiliations:** ^1^ Genomic Research Laboratory, Department of Medicine, Geneva University Hospitals (HUG) and University of Geneva, Geneva, Switzerland; ^2^ Division of Infectious Diseases, Department of Medicine, Geneva University Hospitals (HUG), Geneva, Switzerland; ^3^ Laboratory of Bacteriology, Division of Laboratory Medicine, Department of Diagnostics, Geneva University Hospitals (HUG), Geneva, Switzerland; ^4^ Division of Cardiovascular Surgery, Department of Surgery, Geneva University Hospitals (HUG), Geneva, Switzerland

**Keywords:** cardiac surgery, clinical metagenomics, heart valve, microbiome, next-generation sequencing

## Abstract

**Background:**

Infective endocarditis (IE) is a rare and life-threatening condition with considerable mortality rates. Diagnosis is often complicated by negative blood culture results, limiting the accurate identification of causative pathogens. This study aimed to evaluate the effectiveness of metagenomic next-generation sequencing (mNGS) of cardiac valve specimens compared to conventional clinical laboratory methods for identifying pathogens in IE.

**Methods:**

Nineteen patients with suspected IE who were scheduled for surgical valve removal were prospectively enrolled. The metagenomic workflow included bacterial DNA enrichment from resected valves using the Molzym Ultra-Deep Microbiome Prep, sequencing of metagenomic libraries using the Illumina MiSeq platform, and Kraken 2 taxonomic assignments based on read data.

**Results:**

Valve mNGS achieved a sensitivity of 82.4% and a specificity of 100% relative to the final adjudicated pathogen diagnosis. Blood culture, considered the reference standard, exhibited slightly higher sensitivity (88.2%) with comparable specificity (100%). In comparison, valve culture (sensitivity: 29.4%, specificity: 50.0%) and microscopy (sensitivity: 35.3%, specificity: 100%) showed lower diagnostic performance. Delays between blood culture negativization and valve resection impacted mNGS sensitivity, likely due to pathogen clearance. Notably, valves resected within 12 days from blood culture negativization achieved 100% diagnostic accuracy, emphasizing the importance of timing for optimal mNGS results.

**Conclusion:**

This study underscores mNGS as a valuable diagnostic tool for detecting IE pathogens, complementing traditional diagnostic methods. The detection of antibiotic resistance genes and multi-locus sequence typing profiles in some samples further demonstrated its utility.

## 1 Introduction

Infective endocarditis (IE) represents an inflammation of the inner layer of the heart (endocardium), notably cardiac valves, mostly of bacterial origin. IE is a rare condition, with 1.5–11.6 cases per 100,000 person-years (Bin Abdulhak et al., 2014), but it presents diagnostic and therapeutic challenges, as evidenced by its high mortality rates: 10–24% short-term and 22–37% long-term for 30-day and 1-year mortality, respectively (Ahtela et al., 2019).

Since 2009, there has been an increased incidence of IE in several European countries. Possible contributing factors include ageing population, increased use of cardiovascular devices, increase in intravenous drug use, emergence of coagulase-negative staphylococci and enterococci (not targeted by antibiotic prophylaxis strategies) as causative agents, improved clinical awareness of IE and recent restriction of antibiotic prophylaxis before invasive dental procedures (Chu et al., 2008; Thornhill et al., 2020).

The most common causative agents of IE are gram-positive bacteria–staphylococci, streptococci and enterococci –followed by the gram-negative HACEK group (*Haemophilus* spp., *Aggregatibacter* spp., *Cardiobacterium hominis*, *Eikenella corrodens* and *Kingella* spp.) organisms (Moreillon and Que, 2004). Blood cultures, a gold standard for IE diagnosis, yield negative results in about 20% cases (Kong et al., 2022). Negative results are mainly caused by antibiotic drugs administration prior to the collection of blood for culture as well as infections with difficult-to-culture bacteria such as *Coxiella burnetii*, *Bartonella* spp., *Brucella* spp. and *Tropheryma whipplei*. Yet, the causative agent of culture-negative IE can be identified in 50–80% of cases using alternate diagnostic assays such as serological testing, cardiac valve culture, PCR, qPCR, microscopy or immunochemistry, as well as blood PCR or qPCR assays (Fournier et al., 2010; Fournier et al., 2017).

The current guidelines of the American Association for Thoracic Surgery recommend PCR testing to identify/confirm causative organisms in excised valves (Pettersson and Hussain, 2019). Next-generation sequencing (NGS) techniques, such as targeted 16S rRNA gene amplicon sequencing and metagenomic NGS (mNGS), offer the capability to identify co-pathogens and those with low relative abundance, which might be missed by traditional culture methods and 16S rRNA broad-range PCR (BR-PCR) (Eichenberger et al., 2023; Flurin et al., 2023; Hong et al., 2023). Moreover, mNGS presents the potential for pathogen typing and predicting antibiotic resistance profiles (Cheng et al., 2018; Chan et al., 2019; Cheng et al., 2019; Choutko et al., 2019; Flurin et al., 2023).

The aim of this study was to evaluate the diagnostic performance of mNGS on cardiac valve specimens from 19 patients with suspected IE, comparing it with that of standard clinical laboratory tests. Understanding the strengths and limitations of mNGS is crucial for advancing diagnostic strategies, particularly in cases where conventional methods may not always provide definitive results.

## 2 Methods

### 2.1 Participants

The study was conducted at the University Hospitals of Geneva (HUG), Geneva, Switzerland. Between October 2017 and January 2020, participants with possible infective endocarditis (IE) were recruited as part of this prospective cohort study. Possible IE was defined based on modified Duke’s criteria, requiring either 1 major and 1 minor criterion or 3 minor criteria to be met (Li et al., 2000). All adults (≥18 years old) were eligible for the study if IE was suspected and a surgical intervention was scheduled. Exclusion criteria included hematopoietic stem-cell transplant recipients and possible IE managed without surgical intervention. We examined all microbiological data, including blood and biopsy cultures, biopsy microscopy, and, where relevant, BR-PCR or species-specific qPCR on blood or surgical biopsies, and serological testing. The primary outcome measured was the concordance between mNGS analyses and other microbiological findings, as well as with the retrospectively adjudicated final diagnosis of IE, which was based on an aggregate of clinical and microbiological data. All procedures performed in this study involving human participants were in accordance with the Declaration of Helsinki.

### 2.2 Blood culture

An average of three sets of blood culture bottles (BD BACTEC Standard Anaerobic/Aerobic medium, BD, USA) was filled with 8–10 mL of blood sampled through peripheral venous puncture and incubated into an automated continuous monitoring system (BD BACTEC FX 400, BD) at 35°C for 5 days. Positive blood cultures flagged by the system were then sub-cultured onto the following agar plates: Columbia agar with 5% sheep blood (BioMérieux, France), chocolate agar (BioMérieux), MacConkey agar (BioMérieux), Columbia colistin-nalidixic acid (CNA) agar with 5% sheep blood (BD, USA) and CDC anaerobic blood agar plates (Anaerobe Systems, USA). The solid media were incubated under aerobic conditions (72 h incubation at 35°C in 5% CO_2_ atmosphere) except for CDC agar plates that were incubated under anaerobic conditions (48 h incubation at 35°C). This work was performed either by a lab technician for samples collected before 2019 or by the WASPLab automated system (Copan, Italy) after 2019. The latter concerns 4 study participants, namely number 16, 18, 19 and 20. Each plate was manually assessed for growth and if positive, microorganisms were identified using matrix-assisted laser desorption ionization time-of-flight mass spectrometry (MALDI-TOF MS) BioTyper (Bruker Daltonics, Germany) system and antimicrobial susceptibility testing (AST) was performed subsequently using the disk diffusion method following EUCAST recommendations with a SIRscan image analyzer (i2A, France) (Cherkaoui et al., 2020).

### 2.3 Valve culture

A fragment of the biological valve or of the tissue associated with the mechanical valve was carefully cut into small pieces in a sterile environment (LabGard, NuAire, USA) and placed into a ProbeAX mixing vessel (Axonbiotech, Germany). The tube was then run onto the grinding device (Ultra-TurAX Tube Drive, IKA, Germany) for 3 minutes at 6,000 rpm to homogenize the tissue. At least 3 mL of the resulting homogenized liquid solution was transferred to an emptied eSwab tube (490CE.A, Copan, Italy) and plated manually (before 2019) or via the WASPLab automated system (after 2019) onto: Columbia agar with 5% sheep blood, chocolate agar, MacConkey agar, Columbia colistin-nalidixic acid (CNA) agar with 5% sheep blood and CDC anaerobic blood agar. Additionally, 5 mL of brain-heart infusion (BHI) broth (BD, USA) was inoculated with the homogenized liquid solution. Inoculated solid and liquid media were incubated under aerobic conditions (72 h incubation at 35°C in 5% CO_2_ atmosphere) except for CDC agar plates that were incubated under anaerobic conditions (48 h incubation at 35°C). After visual identification of positive BHI cultures, they were subsequently plated onto solid media for aerobic cultivation, following the procedure described above. Every day, each plate was manually assessed for growth, and if positive, microbes were identified by MALDI-TOF MS and AST performed as described above.

### 2.4 Methicillin-susceptibility testing of *Staphylococcus aureus*

Methicillin-susceptibility for *S. aureus* isolates from valve tissue or blood cultures was assessed both phenotypically by performing AST (see above) and by real-time qPCR targeting *mecA* and *femA* genes as described previously (Francois et al., 2003; Emonet et al., 2016).

### 2.5 Microscopy

Smear slides for microscopic examination were prepared either from a droplet of homogenized liquid valve samples (see above) or from the scrubbed swab in case of mechanical valve samples. Two slides were prepared for each sample and then stained with Color Gram Kit (BioMérieux, France) and Acridine Orange (BD, USA). Results were interpreted as semi-quantitative based on the number of cells counted in a microscopic field at 100×-magnification for bacterial cells (+, 1–5 forms/field; ++, 5–25 forms/field; +++, >25 forms/field) following published international recommendations (Church et al., 2000).

### 2.6 Serological tests for *Bartonella henselae*, *Brucella* spp., *C. burnetii* and *Francisella tularensis*

Serum samples were sent to laboratories accredited for serological analysis to detect antibodies to *B*. *henselae* (Institute of Medical Microbiology, Zurich, Switzerland), *C*. *burnetii* (Central Institute of the Valais Hospital, Sion, Switzerland), *Brucella* spp. (CERBA Laboratory, Cergy-Pontoise, France) and *F*. *tularensis* (Centre for Laboratory Medicine, St. Gallen, Switzerland).

### 2.7 Broad-range 16S rRNA PCR on blood and valve tissues

BD Vacutainer K2 EDTA Tubes (BD, USA), used to collect blood samples from peripheral venous puncture, and frozen stored valve samples were sent to the certified diagnostic laboratory (Institute of Medical Microbiology, Zurich, Switzerland) for BR-PCR testing. DNA extraction and identification by 16S rRNA gene amplification and Sanger sequencing were performed as described previously (Bosshard et al., 2003; Rampini et al., 2011).

### 2.8 Specific qPCR for *C. burnetii* and *T. whipplei* on blood and valve tissues

Blood samples collected through peripheral venous puncture in BD Vacutainer K2 EDTA Tubes (BD, USA), and frozen stored valve samples were sent to the certified diagnostic laboratories for identification of *C*. *burnetii* (Central Institute of the Valais Hospital, Sion, Switzerland) and *T. whipplei* (Institute for Medical and Molecular Diagnostics, Zurich, Switzerland) by qPCR.

### 2.9 DNA extraction from cardiac valves

DNA extraction from cardiac valves was carried out on the material remaining after the removal of a valve fragment for bacterial cultivation. The specimen was minced with a scalpel and homogenized with Hard Tissue Homogenizing Mix 2 mL-tubes (Omni International, USA) on a Bead Ruptor 4 (Omni International) at speed 3 for 30 s. DNA extractions were performed using the Ultra-Deep Microbiome Prep Kit (Molzym, Germany), which is designed to enrich bacterial DNA by selectively lysing host cells and removing human DNA through DNase treatment. Two different pre-treatments were applied to each specimen. The first (hereafter, MOLZ) included proteinase K-based tissue solubilization following the manufacturer’s instructions. The second (hereafter, MOLZ-CH) used a collagenase/hyaluronidase mixture instead. For that purpose, to the valve homogenate we added 1/10 volume of each of the three enzymes: Type I and II collagenases from *Clostridium histolyticum* (Sigma C0130 and C6885), and hyaluronidase from bovine testes (Sigma H3506), prepared as a 2% solution in Dulbecco’s phosphate-buffered saline with MgCl_2_ and CaCl_2_ (DPBS; D8662, Sigma-Aldrich). Reactions were incubated on a thermomixer for 90 min at 37°C with shaking at 1,000 rpm. Purified DNA was eluted in 100 µL of water and stored at –20°C. For negative extraction controls (NECs), DPBS was used instead of clinical samples.

**Supplementary Table S1** provides details regarding the sample mass and variations to the core extraction protocols, such as the addition of a buffer prior or after valve homogenization and the addition of a spin/centrifugation step for removal of macroscopically visible undigested valve material. For patient #16, two available valve fragments were analyzed.

### 2.10 Quantification of human and bacterial DNA

The concentrations of human and bacterial DNA in valve extracts were determined by qPCR targeting human beta-actin and bacterial 16S rRNA reference genes, respectively, as previously described, using 1 µL of non-diluted DNA extract (Leo et al., 2021).

### 2.11 Metagenomic DNA sequencing

Metagenomic libraries were prepared at Fasteris (Plan-Les-Ouates, Switzerland) using Nextera XT DNA Sample Preparation Kit (Illumina, USA) and sequenced (2×250 cycles) on an Illumina MiSeq instrument with the MiSeq Reagent Kit v3.

### 2.12 Bioinformatics analysis

FASTQ files were trimmed with Trimmomatic v.0.36 using the following parameters: LEADING 11, TRAILING 11, SLIDINGWINDOW:20:28 and MINLEN:100. Duplicated reads were removed using the in-house script remove_duplicate_v2.pl (https://github.com/GRL-HUG/duplicates.git). Low-complexity reads were filtered out with Komplexity (–threshold 0.5 –filter) and fastq files were rewritten using Fastq-pair (Clarke et al., 2019; Edwards and Edwards, 2019).

Human DNA sequence clean-up was performed by assigning reads to the human genome (GRCh38.p13) using Kraken 2 in paired-end mode (Wood et al., 2019). Unassigned read pairs were subjected to further analysis using Kraken 2 (–confidence 0.1), in three successive rounds (Lazarevic et al., 2022), utilizing genome databases for bacteria, fungi/viruses/archaea/protozoan parasites, and prokaryotic viruses. At each step, Bracken was employed to re-evaluate the relative abundances of species (Lu et al., 2017). Bracken databases were built with k-mer size of 35 and ideal read length of 250.

For the purpose of comparison, we also performed taxonomic assignments of host-depleted reads using MetaPhlAn 3 (v.3.0.1), using default parameters (Beghini et al., 2021).

Quality filtered non-human reads were assembled using metaSPAdes v.3.12.0 with a k-mer set -k 21,33,55,77 (Nurk et al., 2017). Contigs were tested for the presence of acquired antimicrobial resistance genes (ARG) with ResFinder 4.1 (https://cge.cbs.dtu.dk/services/ResFinder/, accessed 01-09-2022) with ≥90% identity and minimum length of 40% (Bortolaia et al., 2020). To associate ARG to a bacterial host, we performed BLASTN analysis (https://blast.ncbi.nlm.nih.gov/Blast.cgi, accessed 07-09-2022) of relevant contigs (Altschul et al., 1990).

To determine sequence types (ST) of identified IE pathogens, we compared assembled contigs to the PubMLST allele sequence and associated profile data using the https://cge.cbs.dtu.dk/services/MLST/web-server (version 2.0.9, accessed 19-09-2022) (Jolley et al., 2018).

Genetic relatedness between genomic sequences of relevant streptococcal species were assessed using OrthoANI, which calculates average nucleotide identity (ANI) (Lee et al., 2016). From the (100–ANI) distance matrix we constructed the neighbor-joining tree using the *nj* function implemented in R 4.2.0 ape 5.6-2 package (Paradis et al., 2004; R Core Team, 2022).

### 2.13 Statistical analysis

The negative and positive percent agreement (NPA/PPA) between mNGS results and those from blood culture, valve microscopy, and valve culture were calculated using 2×2 contingency tables. Sensitivity, specificity, and accuracy of diagnostic methods were determined using the final diagnosis of IE as a reference. This diagnosis was established through clinical adjudication after a thorough review of each participant’s electronic health records. Given that microscopy alone does not provide species-level identification, the reference cell morphology and staining characteristics of relevant species were considered when comparing valve microscopy results with those from mNGS and the final pathogen diagnosis. A 95% confidence interval was applied to all calculated metrics, and the McNemar test was used for comparison analysis of the obtained results.

Wilcoxon tests were used to compare differences in bacterial load or contaminant abundance.

## 3 Results

### 3.1 Cohort description

From 19 patients suspected of IE who underwent cardiac valve resection, we collected relevant data from routine diagnostic tests, including culture and microscopy, as well as molecular and serology testing if applicable (**Supplementary Table S2**). mNGS analysis for all samples was conducted in a research laboratory (**Supplementary Table S1**). Seventeen patients were ultimately diagnosed with IE (15 males and 2 females), and the diagnosis was rejected in two cases (1 male and 1 female). Patients had a median age of 65 years, with a range of 30–84 years.

### 3.2 Routine clinical microbiology tests

Blood cultures detected bacteria in 15/19 patients initially categorized as having possible or definite IE (**Supplementary Table S2**). In all but one case, a single pathogen was identified, mostly represented by gram-positive cocci belonging to genera *Staphylococcus* (n=5), *Streptococcus* (n=5), *Aerococcus* (n=1) and *Abiotrophia* (n=1). In two cases, the causative pathogen was a gram-negative HACEK organism–*Aggregatibacter aphrophilus* and *C*. *hominis*. In one case, culture identified a mixed infection by *Streptococcus constellatus* and *A*. *aphrophilus*.

Valve biopsy cultures confirmed 4/15 blood-culture positive and 3/4 blood-culture negative results. In one sample, the culture of the biopsy identified *Prevotella oralis* in addition to the two species found by blood culture (*S*. *constellatus* and *A*. *aphrophilus*). For 10 patients with positive blood-cultures, no bacterial growth was observed for valve biopsies samples. Finally, in one patient, valve culture revealed *Staphylococcus epidermidis*, while blood culture remained sterile; however the growth was observed only in liquid medium, suggesting a contamination.

Broad-range 16S rRNA gene testing was performed for patients #7 and #8 on blood and valve samples, respectively. The results were a negative finding for patient #7 and identification of *Streptococcus agalactiae* in patient #8, consistent with the blood culture results.

The blood and valve qPCR test for *T*. *whipplei* (patients #2 and #19) yielded negative results, consistent with findings from other tests. In patient #19, *C*. *burnetii* was detected through blood and valve qPCR and by serology; routine blood and valve culture tests do not identify this intracellular pathogen.

Negative serological tests of five patients were in agreement with other routine laboratory tests. In patient #19, positive *C*. *burnetii* serology was in agreement with the blood qPCR test result. Patient #20 was serologically positive for *B*. *henselae*, while other routine tests pointed to *Streptococcus gallolyticus* as the IE causing pathogen, suggesting a prior *B*. *henselae* infection unrelated to the current IE episode.

Microscopic examination of valves yielded concordant results with valve cultures in 12 out of 19 cases. Among these, 10 cases showed negative findings, while two were positive. In patient #6, the identification of gram-positive cocci and gram-negative rods matched the valve culture; however, the relatively minor presence of gram-positive rods did not align with the organisms isolated in culture. Furthermore, valve microscopy detected the presence of bacteria in three patients where valve cultures were negative. Conversely, in three cases where valve cultures were positive, microscopic examinations did not reveal any bacteria.

### 3.3 Reagent contaminants and criteria for positive mNGS results

**Figure 1:**
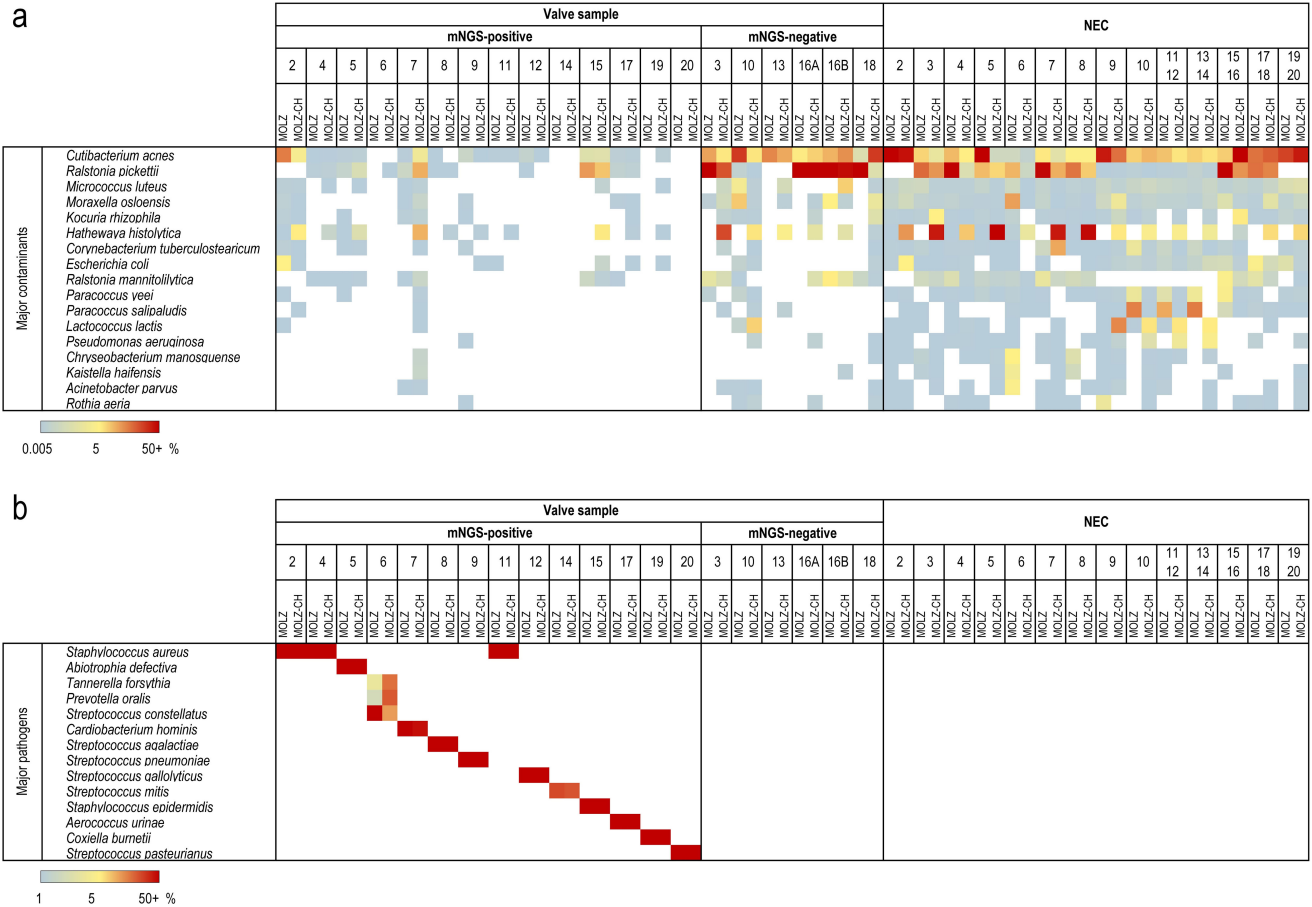
Major contaminant and pathogen species identified in valve samples and negative extraction controls (NEC). Relative abundances determined by Kraken 2 are plotted according to the colour gradient scale given at the bottom. **(a)** Contaminant species. Among 86 putative contaminant species, those with the relative abundances >2.5% in at least one NEC are reported. **(b)** IE-causing pathogens. Relative abundances are reported for the most abundant species of each genus given that they were, in both extracts, >1%, and higher than the summed up relative abundances of contaminant species.

**Figure 2:**
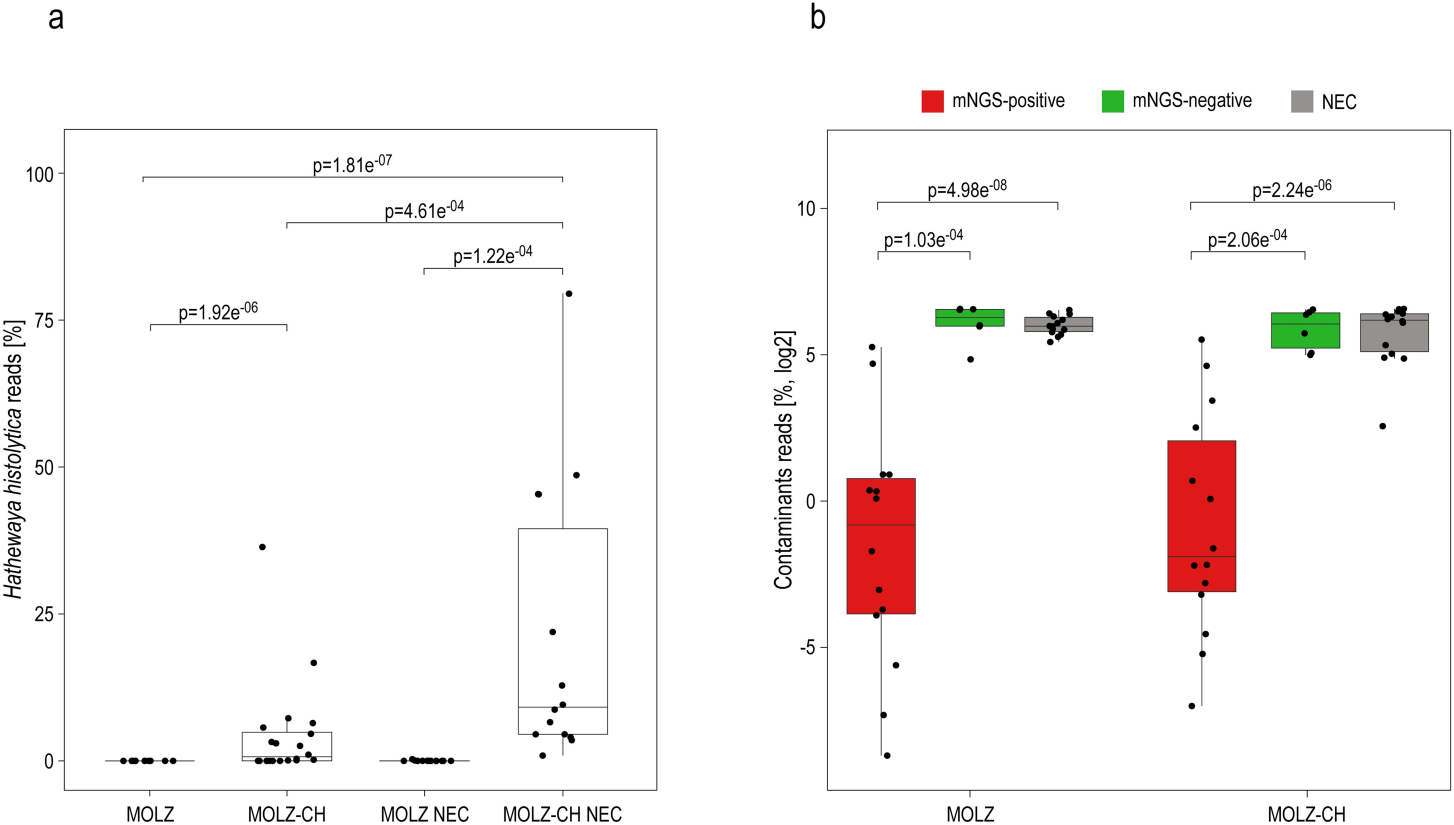
Putative reagent contaminants in the host-depleted mNGS dataset. **(a)** Percentage of reads assigned to *H. histolytica*. The statistical significance of variations related to the extraction method (MOLZ *vs* MOLZ-CH) for valve samples or NECs was evaluated using the Wilcoxon signed-rank test. Differences between valve samples and NECs were compared using the Wilcoxon rank-sum test. Only changes that were statistically significant are reported. **(b)** Percentage of reads assigned to putative contaminant species. The reads assigned to putative contaminant species were summed up and expressed relative to the total number of assigned non-human reads. Wilcoxon rank-sum test was used to assess the differences between mNGS positive valve samples, mNGS-negative valve samples and negative extraction controls (NECs), processed with the same extraction method. Only significant changes are reported.

The mNGS analysis yielded substantially more reads for valve samples compared to NECs (**Supplementary Table S1**). We identified 86 species present in at least half (14/28) of the NECs, with mean relative abundances being higher than those in valve specimens. These species were deemed reagent contaminants (**Figure 1**). *Cutibacterium acnes* was the most abundant contaminant followed by *Ralstonia pickettii*, *Micrococcus luteus*, and *Moraxella osloensis* (**Figure 1**). *Hathewaya histolytica* (*Clostridium histolyticum*) was detected in all MOLZ-CH NECs, at significantly higher abundance compared to MOLZ NECs (**Figure 2**). Obviously, the *H*. *histolytica* DNA originated from the clostridial collagenase preparations used in the MOLZ-CH procedure.

A valve was regarded as infected when the relative abundance of at least one bacterial species in both MOLZ and MOLZ-CH data sets was (i) greater than 1% (**Figure 1**) and (ii) higher than the sum of the relative abundances of contaminant species. If several species of the same genus matched these criteria, the one with the greatest relative abundance was deemed IE-causing. When considering MOLZ- and MOLZ-CH-processed samples separately, additional species that exceeded the positivity cut-off of 1% in one of the two extracts were identified for two valve specimens (#6 and #8) (**Supplementary Table S3**). However, these species had substantially lower abundance compared to the dominant species of the same genus, which did not alter the outcome in identifying the IE-causing pathogen.

The cumulative relative abundance of contaminant species was substantially lower in mNGS-positive samples than in those considered mNGS-negative (**Figure 2**). Taxonomic profiles obtained using Kraken 2 (**Figure 1**) and those generated by MetaPhlAn 3 (**Supplementary Figure S1**), a more specific but less sensitive profiling tool, were highly congruent.

While we found no pathogen in any of the NECs when assessing them against the criteria for mNGS-positivity, three specific NECs exhibited a substantial proportion of reads matching pathogens identified in their corresponding clinical specimens (**Supplementary Figure S2**). These findings strongly suggest the possibility of sample-to-NEC contamination during concurrent processing.

### 3.4 Comparison of mNGS and routine tests results

Overall, valve mNGS and blood culture results (**Supplementary Table S2**) showed high positive percent agreement (PPA) of 85.7% (CI 95% 68.6–94.3%) but moderate-to-low negative percent agreement (NPA) of [40% (CI 95% 14–73.2%), p=0.65]: 11 blood culture-positive and two -negative tests results were confirmed by valve mNGS. In one patient (#6), valve mNGS confirmed the presence of *S. constellatus*, did not confirm *A*. *aphrophilus* but identified two additional bacteria, *Tannerella forsythia* and *P. oralis*; this case was considered ‘polymicrobial IE’ for the purpose of agreement analyses. In three valve mNGS tests, pathogens identified by blood culture were not confirmed. In two cases, valve mNGS identified, respectively, *S*. *aureus* and *C*. *burnetii*, while blood culture detected no growth.

Valve mNGS and microscopy showed low PPA of 42.9% (CI 95% 32.8–53.6%) but 100% (CI 95% 47.8–100%) NPA with five concordant negative results (p=0.0047). In the case of three valves that tested mNGS-positive for streptococci and one for *Staphylococcus*, we confirmed the presence of gram-positive cocci through microscopic analysis. Cocci were also microscopically identified in one valve infected with *Aerococcus urinae*, but only following acridine orange staining. In patient #6 who had a polymicrobial valve infection, staining and microscopy revealed gram-positive cocci and gram-negative rods, consistent with the species identified by valve mNGS. However, gram-positive rods, far less abundant in microscopic observations, did not correspond to species detected by mNGS (and culture). In the remaining eight mNGS-positive valves, no bacteria were identified by microscopic examination.

The comparison of valve mNGS results with those from valve culture (p=0.011) yielded a lower PPA [35.7% (95% CI: 25–48%)] than comparisons with blood culture and microscopy. Notably, mNGS identified a pathogen in nine cases where valve culture was negative. In patient #6, with a mixed infection, both mNGS and culture detected *S*. *constellatus* and *P*. *oralis*, but additional species were identified specifically by each—*T*. *forsythia* by mNGS and *A*. *aphrophilus* by valve culture. The NPA between mNGS and valve culture reached 80% (95% CI: 35.9–96.6%). For patient #18, valve liquid culture was positive for *S*. *epidermidis*, likely indicative of contamination (see above), which would explain the corresponding negative result from mNGS.

### 3.5 Accuracy of diagnostic tests relative to final pathogen identification

In relation to the final diagnosis of IE, valve mNGS demonstrated a sensitivity of 82.4% (CI 95% 56.6–96.2%), specificity of 100% (CI 95% 15.8–100%) and accuracy of 84.2% [(CI 95% 60.4–96.6%), p=0.083] for pathogen identification. This significantly outperformed valve biopsy culture [sensitivity of 29.4% (CI 95% 10.3–56%), specificity of 50% (CI 95% 1.3–98.7%) and accuracy of 31.6% (CI 95% 12.6–56.6%), p=0.022] and microscopy [sensitivity of 35.3% (CI 95% 14.2–61.7%), specificity of 100% (CI 95% 15.8–100%) and accuracy of 42.1% (CI 95% 20.2–66.5%), p=0.00091]. Blood culture showed slightly higher concordance with the final pathogen diagnosis than valve mNGS [sensitivity of 88.2% (CI 95% 63.6–98.6%), specificity of 100% (CI 95% 15.8–100%) and accuracy of 89.5% (CI 95% 66.9–98.7%), p=0.16]. However, the accuracy of mNGS reached 100% (CI 95% 78.2–100%) for valves resected within the first 12 days following blood culture negativization [sensitivity of 100% CI 95% 75.3–100), specificity of 100% (CI 95% 15.8–100%)].

### 3.6 Relative and absolute bacterial load in mNGS-positive and -negative samples

**Figure 3:**
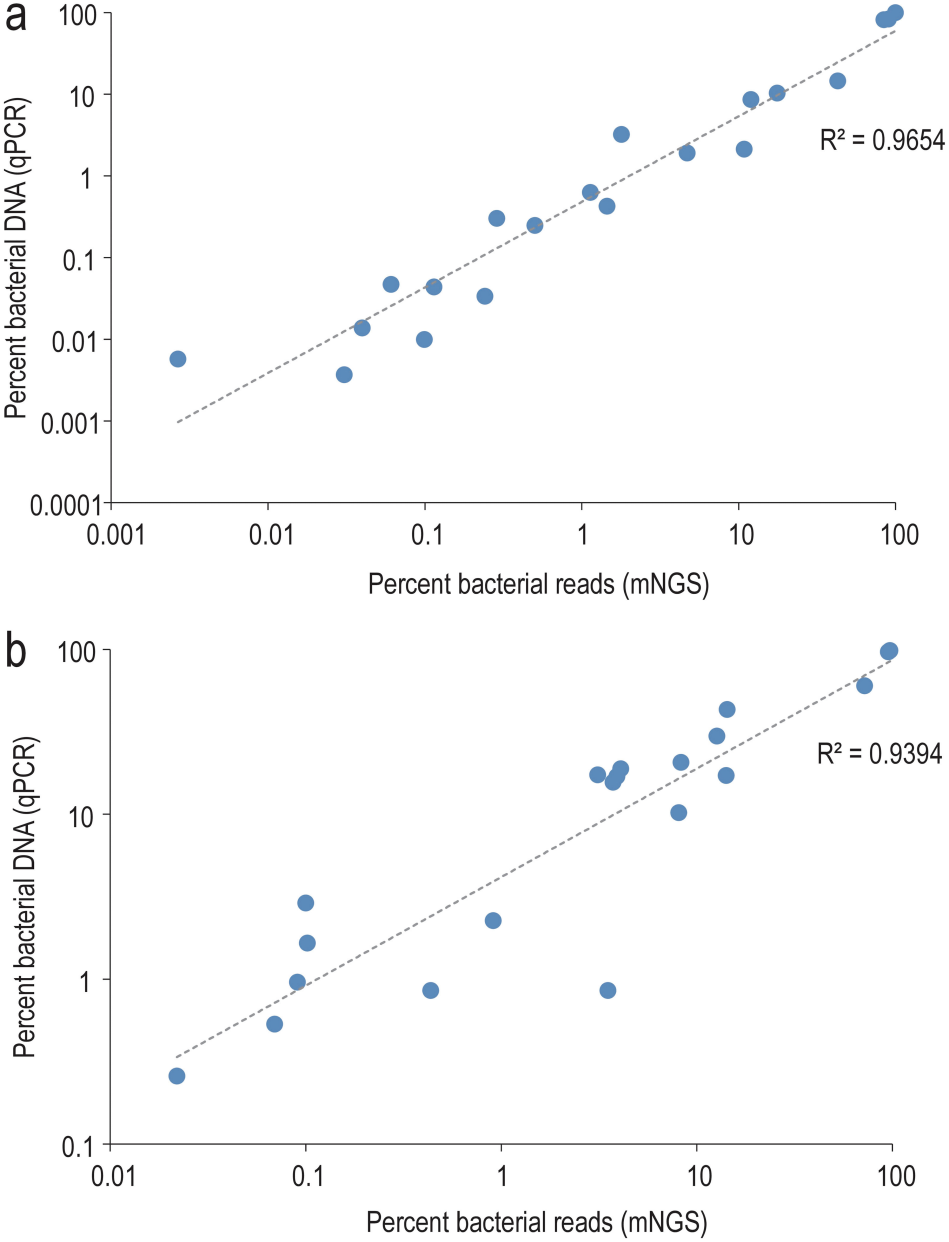
Correlation between qPCR and mNGS-based estimates of the relative bacterial load. The bacterial fraction is expressed relative to the sum of human and bacterial components in MOLZ **(a)** or MOLZ-CH **(b)** samples.

**Figure 4:**
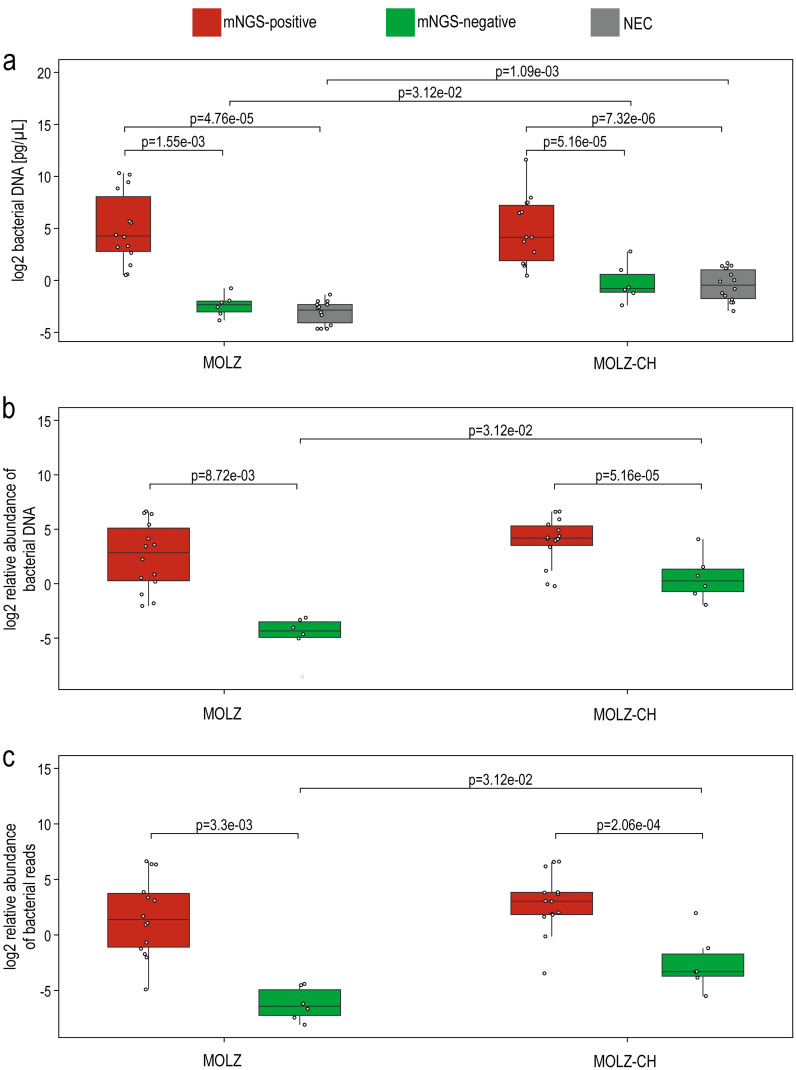
Differences in the bacterial load between samples from mNGS-positive valves, mNGS-negative valves and negative extraction controls. **(a)** qPCR-based assessment of bacterial DNA in the extracts. **(b)** The relative abundance of bacterial DNA, expressed as a percentage of the total DNA content (i.e., the sum of human and bacterial DNA abundances as estimated by qPCR). **(c)** mNGS-based estimate of the relative bacterial DNA abundance, expressed in relation to the combined counts of human and bacterial reads. Statistical significance between sample groups processed with the same extraction method was evaluated using the Wilcoxon rank-sum test, while differences between extraction methods were assessed with the Wilcoxon signed-rank test. Only statistically significant differences are presented. MOLZ and MOLZ-CH denote the two extraction methods used for valve samples, and ‘NEC’ represents negative extraction controls.

Quantification using qPCR revealed significant variations in both bacterial and human DNA extracted across samples, spanning more than three orders of magnitude (**Supplementary Table S1**). qPCR and mNGS provided highly concordant estimates of bacterial DNA proportion relative to the combined bacterial and human DNA components (**Figure 3**). Both absolute and relative abundance of bacterial DNA were notably higher in mNGS-positive samples compared to their mNGS-negative counterparts and NECs (**Figure 4**).

### 3.7 Detection of antibiotic resistance genes and sequence types

The ResFinder analysis of assembled contigs identified ARGs in six samples (**Supplementary Table S4**). In five of them, we were able to reasonably associate the ARG with the detected IE pathogen(s). For example, in patient #6, who had a mixed valve infection, we assigned both tetracycline-resistance *tetM* and macrolide-resistance *ermB* genes to *Streptococcus*, while tetracycline-resistance gene *tetQ* and beta-lactam resistance gene *cfxA3* were assigned to *Prevotella*. Furthermore, we detected the penicillin-resistance conferring beta-lactamase (*blaZ*) gene in two valves (patients #2 and #4) infected by *S*. *aureus*. In the case of patient #4, our mNGS analysis also detected the *S*. *aureus mecA* gene, thus confirming the result of the clinical microbiology laboratory (**Supplementary Table S2**). In two patients (#8 and #20) we confidently associated the identified ARGs with streptococcal IE pathogens. However, in the case of patient #5, who had endocarditis due to *Abiotrophia defectiva*, the two ARGs detected by mNGS, *bla*OXA-60b and *tetM*, were not linked to this species: The *bla*OXA-60b gene was assigned to *Ralstonia*, a major reagent contaminant; the *tetM*-bearing contig was identical or nearly identical to DNA fragments found on plasmids or transposons of various bacterial species.

For five identified IE pathogens (two *Staphylococcus* and three *Streptococcus* species), PubMLST allelic profiles are available. We compared the assembled contigs of samples positive for these species to the reference alleles. Complete MLST profiles were obtained for three samples (**Supplementary Table S5**). For three other, 2–6 (of 7) alleles were identified. In one sample, no MLST loci were detected possibly due to the limited number of pathogen reads.

## 4 Discussion

We show the great potential of the valve mNGS to validate and complement blood culture results in identifying IE causative agents. This was particularly evident when analyzing resected valves within 12 days from the last positive blood culture. Three valves removed 16, 18, and 45 days after the last positive blood culture were considered negative by mNGS, which suggests pathogen clearance most likely due to the antibiotic treatment. However, one valve tested mNGS-positive to *A*. *defectiva* (patient #5) despite an interval of 27 days between the last positive blood culture and valve resection. At the time of valve excision, pathogen cells were likely scarce, dead or non-culturable, leading to negative results in both valve culture and microscopic identifications. However, pathogen DNA was still detectable through mNGS analysis. In line with our findings, Eichenberger et al. (2023) demonstrated that the estimated median duration of IE pathogen detectability following the initiation of antibiotic treatment was 38.1 days for microbial cell-free DNA in plasma, compared to just 3.7 days for blood cultures. Similarly, Flurin et al. (2023) showed that pathogen DNA remained detectable in blood at 100% through 2 days after the last positive blood culture, dropping to 75-79% by one week.

In cases where valve cultures yielded positive results, the samples were collected within a maximum of 7 days following the last positive blood culture. The longest period between the last positive blood culture and positive microscopy findings was 11 days. These findings, along with the predominance of negative results in most specimens, underscore the reduced diagnostic efficacy of valve culture and microscopy when compared to valve mNGS (Shrestha et al., 2015).

Some pathogens identified by valve mNGS but not found by blood culture are more likely to be actual IE causative agents than false positive detections. One of these, *C*. *burnetii* (patient #19), was confirmed by specific blood and valve qPCR testing. In another case (patient #6), *P*. *oralis* and *T*. *forsythia* found by mNGS were missed by blood culture tests, while the valve biopsy culture test did not report *T*. *forsythia*. The combined results of different tests suggest that in this patient, a complex biofilm in the valve tissue was formed of at least four oral species (*S*. *constellatus*, *A*. *aphrophilus*, *T*. *forsythia* and *P*. *oralis*). None of the diagnostics tests used reported all of these species. mNGS analysis reported three of them and *A*. *aphrophilus* was detected but below the chosen positivity threshold.

In the mNGS analysis, we used the relative abundance >1% as an arbitrary threshold for pathogen positivity, provided that it exceeded the summed up relative abundance of contaminant species. When applying a more relaxed mNGS positivity cut-off of >0·04%, pathogens were still not recovered for the three blood-culture-positive patients (#3, #10 and #13). Further relaxing the mNGS positivity criteria by excluding comparison of pathogen to contaminants abundances, led to the identification of *A*. *aphrophilus* in patient #6 and *S*. *aureus* in patient #10. However, this was associated with numerous likely false-positive detections in eight patients, including 17 additional genera per patient.

Significantly higher bacterial loads in mNGS-positive samples when compared to mNGS-negative ones suggests that pan-bacterial qPCR can be an effective initial screening method to select samples for mNGS analysis.

## 5 Limitations

**Figure 5:**
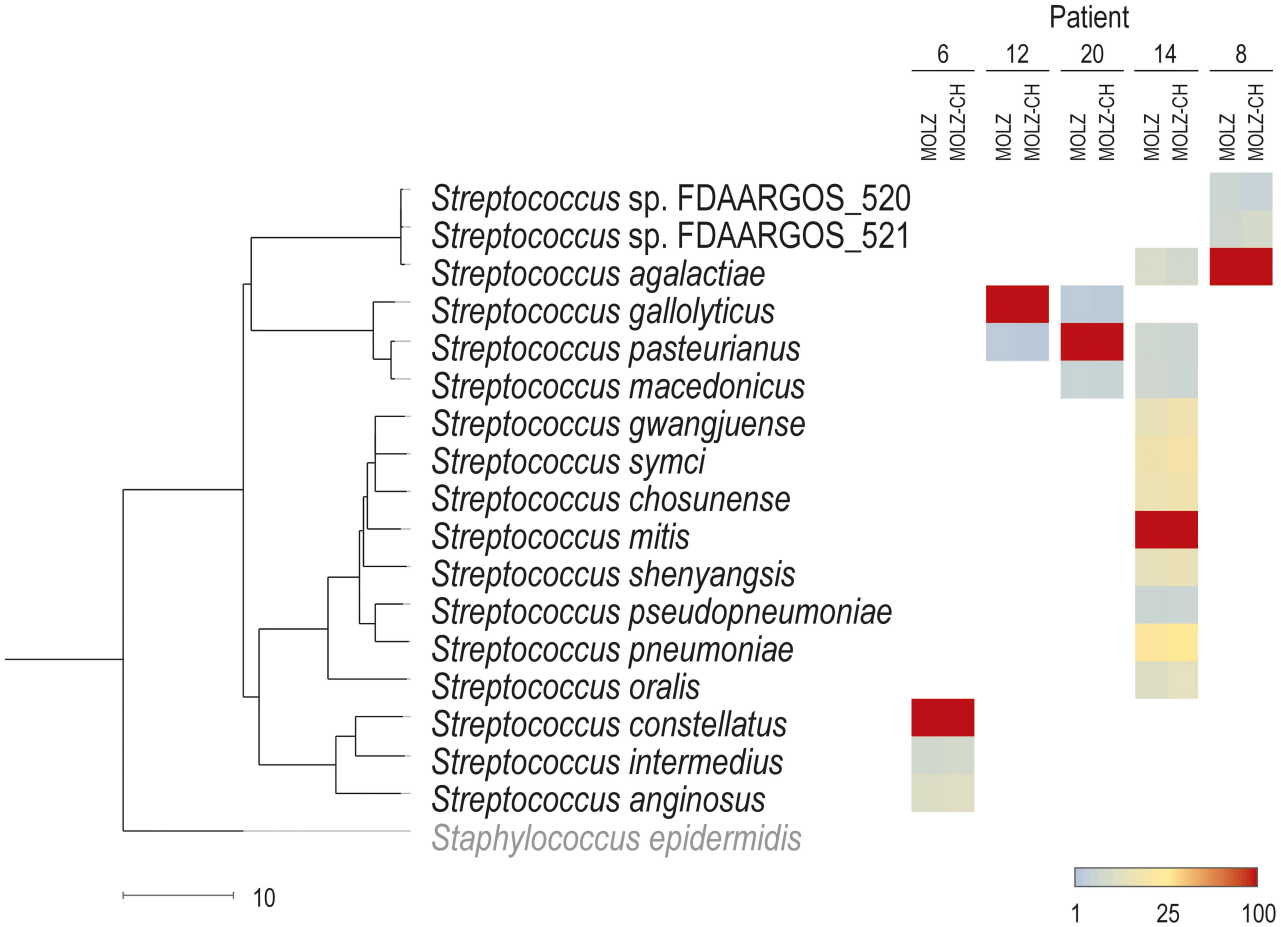
Assignments of reads to streptococcal species. The abundance of the dominant *Streptococcus* species in a given patient is set to 100% for both (MOLZ and MOLZ-CH) valve extracts. The abundance of other streptococcal species (with at least 1% relative abundance in both MOLZ and MOLZ-CH datasets) is expressed as a percentage relative to the dominant species according to the color gradient scale given at the bottom (right). The scale bar (bottom left) shows genetic distance (100–ANI). *S*. *epidermidis* was used as an outgroup to root the dendrogram.

Our study had several limitations. First, the small number of participants may introduce imprecision in performance calculations. Second, our study focused on participants with possible IE scheduled for surgical valve resection, thus representing a selected population. Third, diagnosing IE is challenging and relies on a combination of medical history, clinical signs, imaging, and laboratory tests with imperfect performances, leading to not infrequent equivocal cases. Fourth, the processing of cardiac valve tissue by clinical laboratories is prone to bias when only small fragments are analyzed. Fifth, although mNGS retains some ability to detect pathogens despite antimicrobial therapy, its performance is affected by longer intervals between the suspicion of diagnosis and surgery. Sixth, the accurate discrimination of closely-related bacterial species proves challenging due to short NGS read length and k-mer-based taxonomic classification. In our study, in 13 out of 14 patients with mNGS-positive valve results, the dominant species identified by mNGS was consistent with blood culture/qPCR results. In five of these patients, we detected several streptococcal species with a relative abundance exceeding 1%. **Figure 5** shows a trend of decreasing relative abundances of likely misclassified species as their genetic distance from the major pathogen increases. Seventh, the efficacy of host DNA removal, aimed at increasing the proportion of bacterial-origin reads varied among samples, resulting in different performances in detecting ARGs and bacterial typing. Despite its limitations, mNGS shows promising results when combined with current IE diagnostic approaches.

## 6 Conclusion

Identification of IE pathogens by mNGS of valve specimens is in good agreement with results of blood culture and outperforms valve culture and microscopy. The challenge of identifying IE causative agents via valve mNGS, when valve resection occurs a longer time after blood culture positivity, can be attributed to pathogen clearance. Overall, our results support the utility of mNGS in diagnosing IE, offer insights into its strengths and limitations and pave the way for improved strategies for identifying the causative agents of IE.

## Data Availability

The datasets presented in this study can be found in online repositories. The names of the repository/repositories and accession number(s) can be found below: https://www.ebi.ac.uk/ena, PRJEB57944.

## References

[B1] Ahtela E. Oksi J. Sipilä J. Rautava P. Kytö V. (2019). Occurrence of fatal infective endocarditis: a population-based study in Finland. BMC Infect. Dis. 19, 987. doi: 10.1186/s12879-019-4620-0 31752727 PMC6873758

[B2] Altschul S. F. Gish W. Miller W. Myers E. W. Lipman D. J. (1990). Basic local alignment search tool. J. Mol. Biol. 215, 403–410. doi: 10.1006/jmbi.1990.9999 2231712

[B3] Beghini F. McIver L. J. Blanco-Míguez A. Dubois L. Asnicar F. Maharjan S.. (2021). Integrating taxonomic, functional, and strain-level profiling of diverse microbial communities with bioBakery 3. Elife 10, e65088. doi: 10.7554/eLife.65088 33944776 PMC8096432

[B4] Bin Abdulhak A. A. Baddour L. M. Erwin P. J. Hoen B. Chu V. H. Mensah G. A.. (2014). Global and regional burden of infective endocarditis, 1990-2010: a systematic review of the literature. Glob Heart. 9, 131–143. doi: 10.1016/j.gheart.2014.01.002 25432123

[B5] Bortolaia V. Kaas R. S. Ruppe E. Roberts M. C. Schwarz S. Cattoir V.. (2020). ResFinder 4.0 for predictions of phenotypes from genotypes. J. Antimicrob. Chemother. 75, 3491–3500. doi: 10.1093/jac/dkaa345 32780112 PMC7662176

[B6] Bosshard P. P. Kronenberg A. Zbinden R. Ruef C. Bottger E. C. Altwegg M. (2003). Etiologic diagnosis of infective endocarditis by broad-range polymerase chain reaction: a 3-year experience. Clin. Infect. Dis. 37, 167–172. doi: 10.1086/375592 12856207

[B7] Chan W. S. Au C. H. Leung H. C. Ho D. N. Li D. Chan T. L.. (2019). Potential utility of metagenomic sequencing for improving etiologic diagnosis of infective endocarditis. Future Cardiol. 15, 411–424. doi: 10.2217/fca-2018-0088 31691592

[B8] Cheng J. Hu H. Fang W. Shi D. Liang C. Sun Y.. (2019). Detection of pathogens from resected heart valves of patients with infective endocarditis by next-generation sequencing. Int. J. Infect. Dis. 83, 148–153. doi: 10.1016/j.ijid.2019.03.007 30926543

[B9] Cheng J. Hu H. Kang Y. Chen W. Fang W. Wang K.. (2018). Identification of pathogens in culture-negative infective endocarditis cases by metagenomic analysis. Ann. Clin. Microbiol. Antimicrob. 17, 43. doi: 10.1186/s12941-018-0294-5 30567558 PMC6300891

[B10] Cherkaoui A. Renzi G. Fischer A. Azam N. Schorderet D. Vuilleumier N.. (2020). Comparison of the Copan WASPLab incorporating the BioRad expert system against the SIRscan 2000 automatic for routine antimicrobial disc diffusion susceptibility testing. Clin. Microbiol. Infect. 26, 619–625. doi: 10.1016/j.cmi.2019.11.008 31733376

[B11] Choutko V. Lazarevic V. Gaïa N. Girard M. Renzi G. Leo S.. (2019). Rare case of community-acquired endocarditis caused by Neisseria meningitidis assessed by clinical metagenomics. Front. Cardiovasc. Med. 6112. doi: 10.3389/fcvm.2019.00112 PMC6691042 31448292

[B12] Chu V. H. Woods C. W. Miro J. M. Hoen B. Cabell C. H. Pappas P. A.. (2008). Emergence of coagulase-negative staphylococci as a cause of native valve endocarditis. Clin. Infect. Dis. 46, 232–242. doi: 10.1086/524666 18171255

[B13] Church D. Melnyk E. Unger B. (2000). Quantitative gram stain interpretation criteria used by microbiology laboratories in Alberta, Canada. J. Clin. Microbiol. 38, 4266–4268. doi: 10.1128/jcm.38.11.4266-4268.2000 11060107 PMC87580

[B14] Clarke E. L. Taylor L. J. Zhao C. Connell A. Lee J. J. Fett B.. (2019). Sunbeam: an extensible pipeline for analyzing metagenomic sequencing experiments. Microbiome 7, 46. doi: 10.1186/s40168-019-0658-x 30902113 PMC6429786

[B15] Edwards J. A. Edwards R. A. (2019). Fastq-pair: efficient synchronization of paired-end fastq files. bioRxiv, 552885 [preprint]. doi: 10.1101/552885

[B16] Eichenberger E. M. Degner N. Scott E. R. Ruffin F. Franzone J. Sharma-Kuinkel B.. (2023). Microbial cell-free DNA identifies the causative pathogen in infective endocarditis and remains detectable longer than conventional blood culture in patients with prior antibiotic therapy. Clin. Infect. Dis. 76, e1492–ee500. doi: 10.1093/cid/ciac426 35684984 PMC10169441

[B17] Emonet S. Charles P. G. Harbarth S. Stewardson A. J. Renzi G. Uckay I.. (2016). Rapid molecular determination of methicillin resistance in staphylococcal bacteraemia improves early targeted antibiotic prescribing: a randomized clinical trial. Clin. Microbiol. Infect. 22, 946 e9–94 e15. doi: 10.1016/j.cmi.2016.07.022 27475737

[B18] Flurin L. Fisher C. R. Wolf M. J. Pritt B. S. DeSimone D. C. Patel R. (2023). Comparison of blood-based shotgun and targeted metagenomic sequencing for microbiological diagnosis of infective endocarditis. Open Forum Infect. Dis. 10, ofad546. doi: 10.1093/ofid/ofad546 38075017 PMC10709542

[B19] Fournier P.-E. Gouriet F. Casalta J.-P. Lepidi H. Chaudet H. Thuny F.. (2017). Blood culture-negative endocarditis: Improving the diagnostic yield using new diagnostic tools. Med. (Baltimore). 96, e8392. doi: 10.1097/md.0000000000008392 PMC5708915 29381916

[B20] Fournier P. E. Thuny F. Richet H. Lepidi H. Casalta J. P. Arzouni J. P.. (2010). Comprehensive diagnostic strategy for blood culture-negative endocarditis: a prospective study of 819 new cases. Clin. Infect. Dis. 51, 131–140. doi: 10.1086/653675 20540619

[B21] Francois P. Pittet D. Bento M. Pepey B. Vaudaux P. Lew D.. (2003). Rapid detection of methicillin-resistant Staphylococcus aureus directly from sterile or nonsterile clinical samples by a new molecular assay. J. Clin. Microbiol. 41, 254–260. doi: 10.1128/jcm.41.1.254-260.2003 12517857 PMC149566

[B22] Hong H. L. Flurin L. Greenwood-Quaintance K. E. Wolf M. J. Pritt B. S. Norgan A. P.. (2023). 16S rRNA gene PCR/sequencing of heart valves for diagnosis of infective endocarditis in routine clinical practice. J. Clin. Microbiol. 61, e0034123. doi: 10.1128/jcm.00341-23 37436146 PMC10446860

[B23] Jolley K. A. Bray J. E. Maiden M. C. J. (2018). Open-access bacterial population genomics: BIGSdb software, the PubMLST.org website and their applications. Wellcome Open Res. 3, 124. doi: 10.12688/wellcomeopenres.14826.1 30345391 PMC6192448

[B24] Kong W. K. F. Salsano A. Giacobbe D. R. Popescu B. A. Laroche C. Duval X.. (2022). Outcomes of culture-negative vs. culture-positive infective endocarditis: the ESC-EORP EURO-ENDO registry. Eur. Heart J. 43, 2770–2780. doi: 10.1093/eurheartj/ehac307 35695691 PMC9459867

[B25] Lazarevic V. Gaïa N. Girard M. Mauffrey F. Ruppé E. Schrenzel J. (2022). Effect of bacterial DNA enrichment on detection and quantification of bacteria in an infected tissue model by metagenomic next-generation sequencing. ISME Commun. 2, 122. doi: 10.1038/s43705-022-00208-2 37938717 PMC9792467

[B26] Lee I. Ouk Kim Y. Park S. C. Chun J. (2016). OrthoANI: An improved algorithm and software for calculating average nucleotide identity. Int. J. Syst. Evol. Microbiol. 66, 1100–1103. doi: 10.1099/ijsem.0.000760 26585518

[B27] Leo S. Lazarevic V. von Dach E. Kaiser L. Prendki V. Schrenzel J.. (2021). Effects of antibiotic duration on the intestinal microbiota and resistome: The PIRATE RESISTANCE project, a cohort study nested within a randomized trial. EBioMedicine 71, 103566. doi: 10.1016/j.ebiom.2021.103566 34492446 PMC8426194

[B28] Li J. S. Sexton D. J. Mick N. Nettles R. Fowler V. G. Jr. Ryan T.. (2000). Proposed modifications to the Duke criteria for the diagnosis of infective endocarditis. Clin. Infect. Dis. 30, 633–638. doi: 10.1086/313753 10770721

[B29] Lu J. Breitwieser F. P. Thielen P. Salzberg S. L. (2017). Bracken: estimating species abundance in metagenomics data. PeerJ Comput. Sci. 3, e104. doi: 10.7717/peerj-cs.104 PMC12016282 40271438

[B30] Moreillon P. Que Y. A. (2004). Infective endocarditis. Lancet 363, 139–149. doi: 10.1016/s0140-6736(03)15266-x 14726169

[B31] Nurk S. Meleshko D. Korobeynikov A. Pevzner P. A. (2017). metaSPAdes: a new versatile metagenomic assembler. Genome Res. 27, 824–834. doi: 10.1101/gr.213959.116 28298430 PMC5411777

[B32] Paradis E. Claude J. Strimmer K. (2004). APE: analyses of phylogenetics and evolution in R language. Bioinformatics 20, 289–290. doi: 10.1093/bioinformatics/btg412 14734327

[B33] Pettersson G. B. Hussain S. T. (2019). Current AATS guidelines on surgical treatment of infective endocarditis. Ann. Cardiothorac. Surg. 8, 630–644. doi: 10.21037/acs.2019.10.05 31832353 PMC6892713

[B34] Rampini S. K. Bloemberg G. V. Keller P. M. Buchler A. C. Dollenmaier G. Speck R. F.. (2011). Broad-range 16S rRNA gene polymerase chain reaction for diagnosis of culture-negative bacterial infections. Clin. Infect. Dis. 53, 1245–1251. doi: 10.1093/cid/cir692 21976460

[B35] R Core Team (2022). R: a language and environment for statistical computing (Vienna, Austria: R Foundation for Statistical Computing). Available at: https://www.R-project.org/.

[B36] Shrestha N. K. Ledtke C. S. Wang H. Fraser T. G. Rehm S. J. Hussain S. T.. (2015). Heart valve culture and sequencing to identify the infective endocarditis pathogen in surgically treated patients. Ann. Thorac. Surg. 99, 33–37. doi: 10.1016/j.athoracsur.2014.07.028 25442997

[B37] Thornhill M. H. Dayer M. J. Nicholl J. Prendergast B. D. Lockhart P. B. Baddour L. M. (2020). An alarming rise in incidence of infective endocarditis in England since 2009: why? Lancet 395, 1325–1327. doi: 10.1016/s0140-6736(20)30530-4 32334690

[B38] Wood D. E. Lu J. Langmead B. (2019). Improved metagenomic analysis with Kraken 2. Genome Biol. 20, 257. doi: 10.1186/s13059-019-1891-0 31779668 PMC6883579

