# Supplementary material for: Identification of causative agents of infective endocarditis by metagenomic next-generation sequencing of resected valves
Source: Front Cell Infect Microbiol. 2025 Mar 13;15:1532257. doi: 10.3389/fcimb.2025.1532257 (PMC11966046; doi:10.3389/fcimb.2025.1532257)

Supplementary Figure 1. Microbial species identified by MetaPhlAn 3 analysis in valve samples and negative extraction controls. Relative abundances (percentage) are plotted according to the colour gradient scale given at the bottom. For each clinical or control sample, only species identified in both extracts are plotted. mNGS-positivity/negativity is defined by the Kraken 2 analysis.

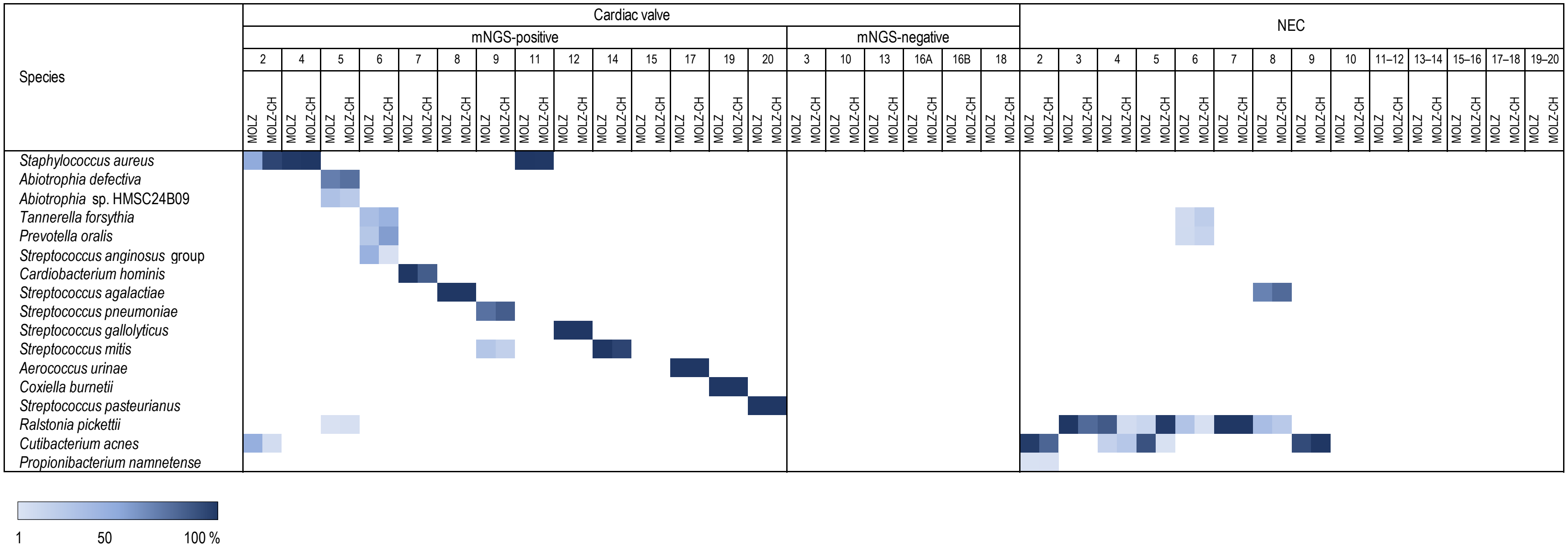

Supplement: Supplementary file 1 [file DataSheet1.pdf]
