# Supplementary material for: Identification of causative agents of infective endocarditis by metagenomic next-generation sequencing of resected valves
Source: Front Cell Infect Microbiol. 2025 Mar 13;15:1532257. doi: 10.3389/fcimb.2025.1532257 (PMC11966046; doi:10.3389/fcimb.2025.1532257)

**Supplementary Figure 2. Simultaneous pathogen identification in valve specimens and corresponding negative extraction controls, indicative of potential contamination from clinical samples to controls.**

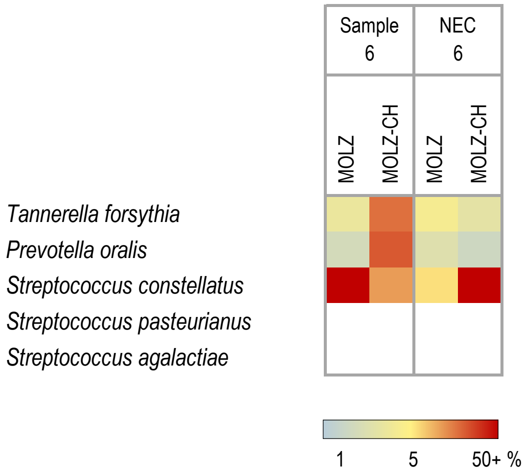

Supplement: Supplementary file 2 [file DataSheet2.pdf]
